# Combined BRAF/MEK inhibition for BRAF-mutant melanoma brain metastases in pregnancy: A case report

**DOI:** 10.3892/ol.2025.15361

**Published:** 2025-10-24

**Authors:** Jaroslav Melus, Michaela Jezberova, Silvia Mikulajova, Katarina Komaromy Gerincova, Martin Karlik, Igor Straka, Marek Krivosik, Peter Valkovic, Gabriela Timarova

**Affiliations:** 1 2nd Department of Neurology, Comenius University Bratislava, Faculty of Medicine, University Hospital Bratislava, Bratislava 833 05, Slovakia; 2 Department of Magnetic Resonance Imaging, Dr. Magnet Ltd., Bratislava 833 05, Slovakia; 3 Department of Neonatology, University Hospital Bratislava, Bratislava 833 05, Slovakia; 4 1st Department of Obstetrics and Gynecology, Slovak Medical University, University Hospital Bratislava, Bratislava 833 05, Slovakia; 5 Institute of Normal and Pathological Physiology, Centre of Experimental Medicine, Slovak Academy of Sciences, Bratislava 813 71, Slovakia

**Keywords:** PAM, brain MTS, neurosurgery, BRAF V600E mutation, D + T

## Abstract

Melanoma is a highly aggressive malignancy with the potential to metastasize to the placenta and fetus. Pregnancy-associated melanoma (PAM) complicated by brain metastases (MTS) is exceedingly rare, and its management requires balancing maternal survival with fetal safety. A 35-year-old pregnant woman with a history of superficial spreading melanoma presented at 24 weeks of gestation with multiple brain MTS. Following multidisciplinary consultation and after obtaining informed consent, two symptomatic brain MTS were surgically resected during pregnancy. Histopathology and immunohistochemistry confirmed metastatic melanoma, and molecular testing identified a B-Raf proto-oncogene serine/threonine kinase V600E mutation. Due to disease progression, targeted therapy with dabrafenib and trametinib was initiated in the third trimester. At 33 weeks of gestation, an acute cesarean section was performed following a focal impaired consciousness seizure. A premature male neonate was delivered, requiring respiratory support and intensive care. Following stabilization, the growth and psychomotor development of the neonate remained normal at follow-up. Postpartum, the mother transitioned to immune checkpoint inhibitor therapy and remains alive with partial remission 16 months after the diagnosis of brain MTS. The present case demonstrates the feasibility of combined neurosurgical and targeted therapeutic approaches for PAM with brain MTS. The case provides valuable clinical and ethical insights into individualized decision-making when managing advanced melanoma during pregnancy, a scenario that remains rare and poorly documented.

## Introduction

Melanoma is a highly aggressive malignancy arising from the malignant transformation of melanocytes, neural crest-derived cells located in the basal epidermis (1). Cutaneous melanoma (CM) is the predominant subtype, accounting for >90% of new melanoma diagnoses and ~75% of skin cancer-associated mortalities due to its rapid systemic dissemination and high metastatic potential (2,3). In autopsy studies, brain metastases (MTS) have been observed in up to 74% of patients with advanced melanoma (4).

Pregnancy-associated melanoma (PAM) is rare, with an estimated incidence rate ranging from 1.4 to 8.5 cases per 100,000 pregnancies, based on data from European and U.S. studies (5–8). Melanoma is also one of the few cancer types capable of metastasizing to the placenta and fetus, accounting for approximately one-third of all reported placental MTS (9). With placental involvement, the transmission risk to the fetus is ~22%, placing such neonates at high risk (10).

The therapeutic landscape of advanced melanoma has been transformed by targeted inhibitors of the mitogen-activated protein kinase (MAPK) pathway and immune checkpoint blockade (11–13). However, systemic treatment during pregnancy remains challenging due to concerns regarding teratogenicity and limited available data. Only a few case reports have described the use of the B-Raf proto-oncogene serine/threonine kinase (BRAF) inhibitor vemurafenib during pregnancy (14–17); however, to the best of our knowledge, the present study is the first report documenting combined BRAF and mitogen-activated protein kinase kinase (MEK) inhibition with dabrafenib and trametinib (D + T) in a pregnant patient with melanoma brain MTS.

The current report presents the clinical course of a 35-year-old woman diagnosed at 24 weeks of gestation with multiple brain MTS from melanoma, who was managed with a multidisciplinary approach, including neurosurgery, targeted therapy and subsequent immunotherapy.

## Case report

### Patient history

The current study presents the case of a 35-year-old pregnant woman with a history of superficial spreading melanoma on the scalp. The primary tumor showed ulceration, vertical growth phase, Clark's level IV (18) and a Breslow thickness of 1.1 mm (19). Initial treatment consisted of an excisional biopsy with narrow resection margins (May 2021). Despite medical recommendations, a radical re-excision of the tumor bed and sentinel lymph node biopsy were not performed, mainly due to circumstances related to the SARS-CoV-2 pandemic.

At the follow-up in January 2023, a whole-body ^18^F-FDG PET/CT revealed a non-specific pulmonary nodule in the left anterior segment (S3) of the lung, without evidence of MTS. The melanoma was staged as IIA (T2bN0M0) according to the American Joint Committee on Cancer system (20). Systemic treatment was not indicated, and routine surveillance was continued.

### Presentation during pregnancy

In January 2024, ~3 years later, the patient presented to the Academician Ladislav Dérer Hospital (University Hospital Bratislava; Bratislava, Slovakia) at 24+2 weeks of gestation with a brief episode of expressive aphasia that resolved within 15 min. Upon admission, the neurological examination was normal.

**Figure 1.**
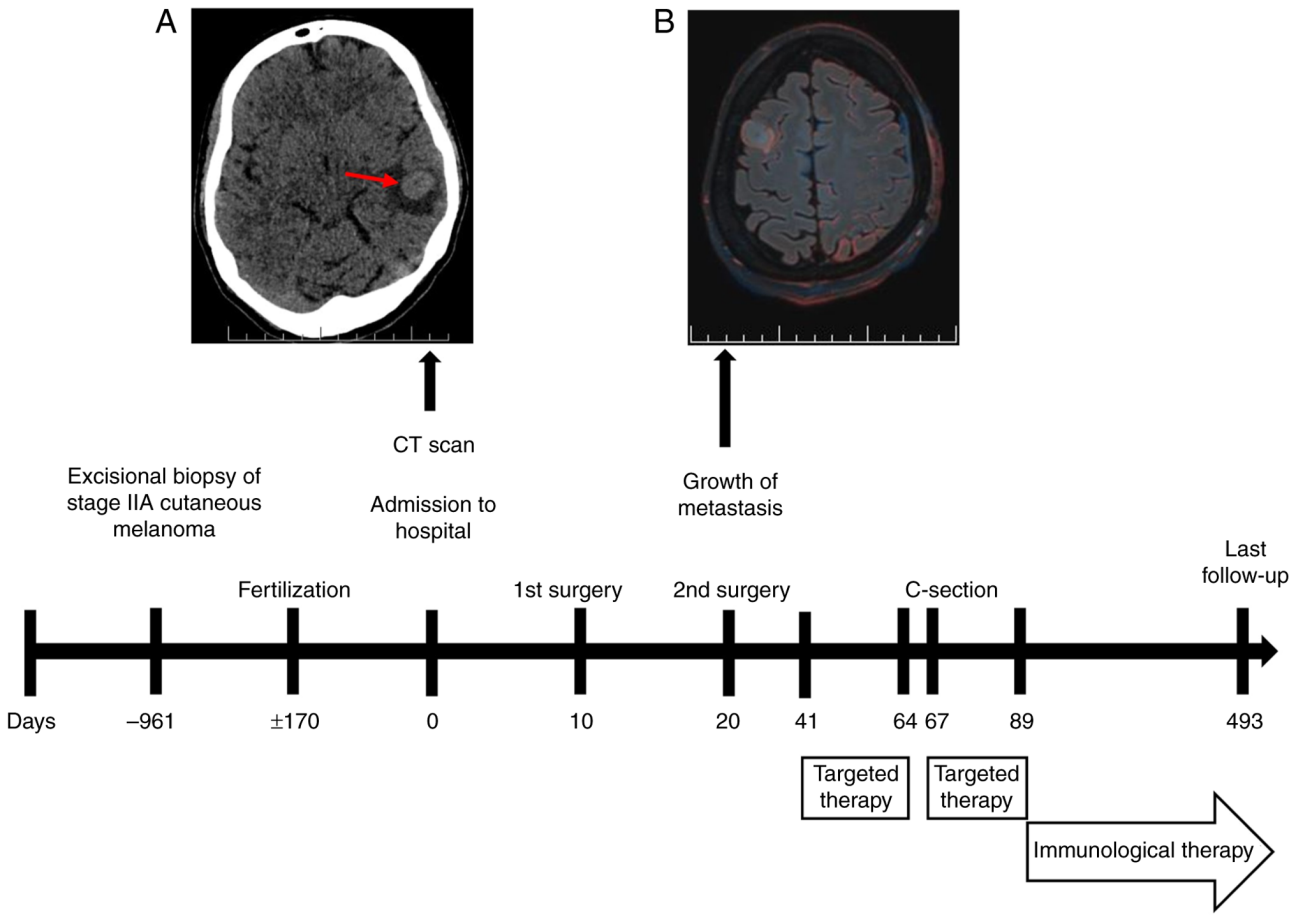
Timeline of the clinical course. (A) Left temporal lobe metastasis with perifocal edema (red arrow; CT axial plane). (B) MRI longitudinal co-registered fusion of 3D fluid-attenuated inversion recovery images showing rapid enlargement of the right frontal metastasis (comparison of images a week apart in January 2024). Scale bars, 10 mm.

**Figure 2.**
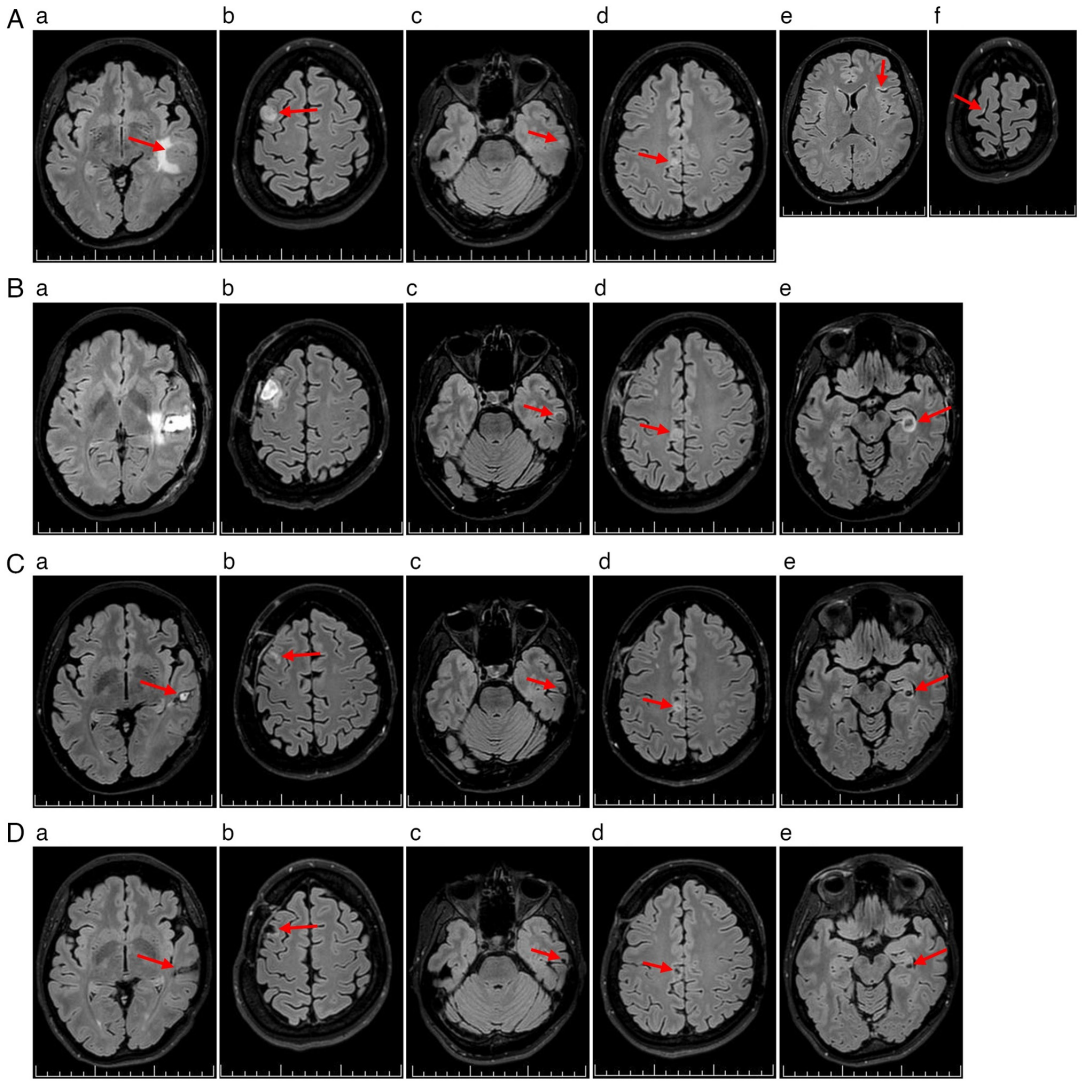
Timeline of the radiological course (MRI fluid-attenuated inversion recovery sequences). (Aa-Ad) Initial images showing four malignant melanoma brain metastases (red arrows). (Ae and Af) Images showing two suspected brain metastases (red arrows). (Ba and Bb) MRI 2 days after surgical resections. (Bc and Bd) Enlargement of the two remaining metastases (red arrows). (Be) Growth of a new metastasis in the dominant hippocampal region (red arrow). (Ca and Cb) Favorable postoperative changes (red arrows). (Cc-Ce) Notable reduction in metastatic lesion volume (red arrows). (Da and Db) Further favorable postoperative changes without signs of recurrence (red arrows). (Dc-De) Partial disease remission (red arrows). Scale bars, 10 mm.

A non-contrast low-dose CT scan of the brain revealed an intra-axial lesion in the left temporal lobe, with perifocal edema (Fig. 1A). The following day, non-contrast 3T brain MRI identified six supratentorial lesions. A total of four lesions showed radiological features consistent with MTS (Fig. 2Aa-Ad), while two small cortical hyperintensities on fluid-attenuated inversion recovery sequences were atypical but suspicious for metastatic disease (Fig. 2Ae and Af).

**Figure 3.**
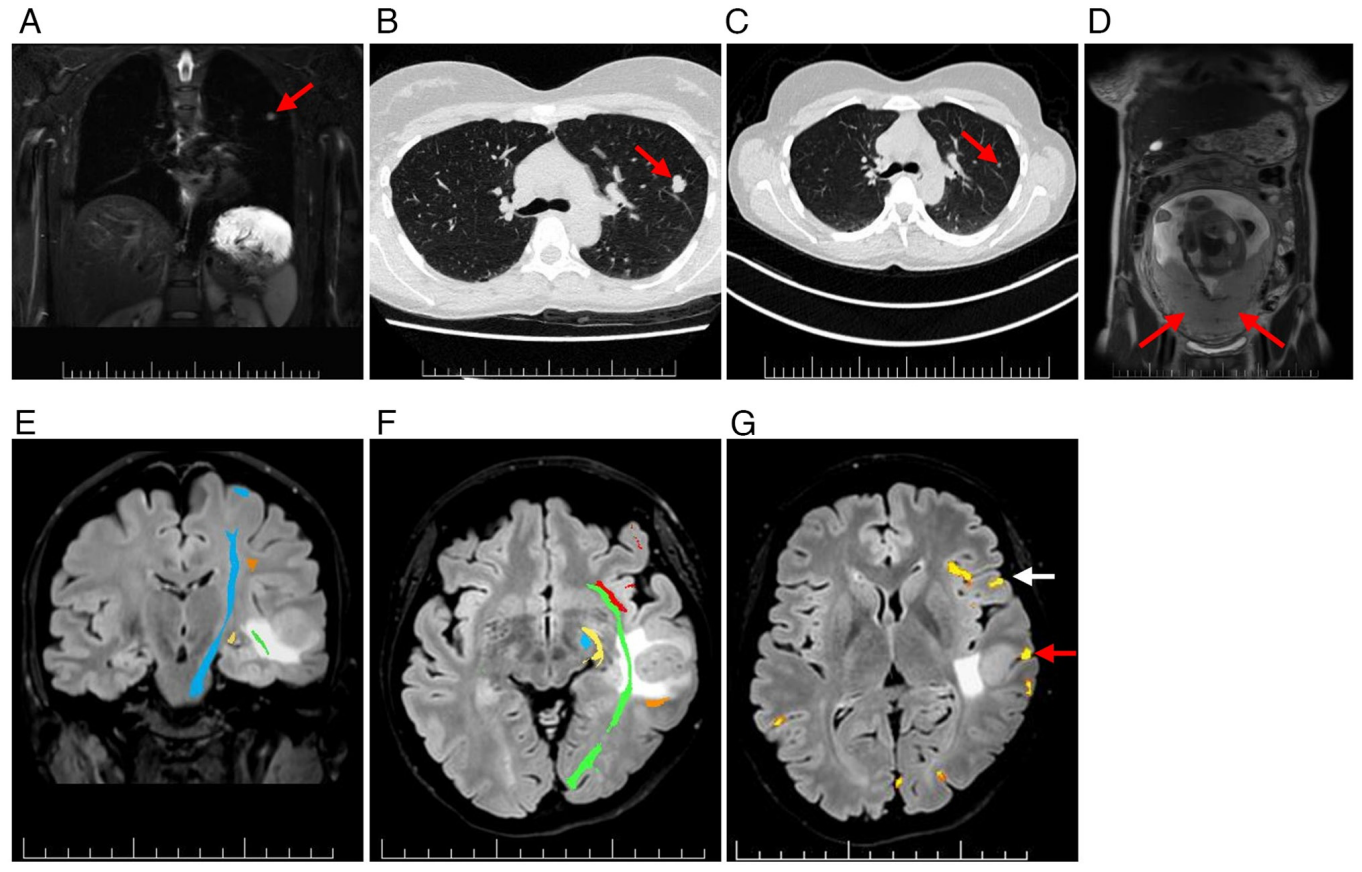
WB-MRI, HRCT pulmonary scans and pre-surgical brain mapping in relation to the left temporal metastasis. (A) WB-MRI confirming the pulmonary nodule in the left upper lobe (red arrow). (B) HRCT scan showing a lobulated pulmonary nodule in the same region (red arrow; January 2024). (C) Pulmonary nodule visible on CT in January 2023 (red arrow). (D) Placenta with homogeneous appearance and no evidence of metastatic involvement (red arrows; coronal T2-weighted image). (E) Diffusion tensor imaging demonstrating major white matter tracts in proximity to the lesion: Corticospinal tract (blue), inferior fronto-occipital fasciculus (green), arcuate fasciculus (orange) and optic radiation (yellow). (F) Tractography showing all major tracts, including the uncinate fasciculus (red). (G) Functional MRI demonstrating eloquent cortical areas relevant to speech (yellow); Wernicke's area (red arrow), Broca's area (white arrow). Scale bars, 10 mm. WB, whole-body; HRCT, high-resolution computed tomography.

For systemic staging, whole-body (WB)-MRI with diffusion-weighted imaging demonstrated a small solid lesion in the left upper lung lobe (Fig. 3A). High-resolution CT confirmed a lobulated nodule in the left S3 region (Fig. 3B). Retrospective comparison with a CT scan from January 2023, showed interval growth of 12 mm (Fig. 3C). No uterine or placental involvement was detected using WB-MRI (Fig. 3D).

### Neurosurgical management

After multidisciplinary counseling and obtaining informed consent, a pro-fetus strategy was adopted. Due to symptomatic peritumoral edema from brain MTS, the patient received intravenous dexamethasone (4 mg every 8 h for 12 doses; total, 48 mg), followed by oral methylprednisolone (16 mg every 8 h), which was gradually reduced and discontinued after 6 weeks.

Preoperative MRI mapping with diffusion tensor imaging and functional MRI showed the largest left temporal MTS abutting eloquent language and motor areas (Fig. 3E-G). In January 2024, an awake craniotomy with resection of the left middle temporal gyrus MTS was performed (Fig. 2Ba). Histopathological analysis of formalin-fixed and paraffin-embedded samples confirmed metastatic melanoma (Fig. S1) (21). Immunohistochemistry demonstrated the following results: Cytokeratin AE1/3(−), S100(+), SOX10(+), melan-A(+), HMB-45(+), preferentially expressed antigen in melanoma(+), with a Ki-67 proliferation index up to 30% (Fig. S1) (22,23). Molecular testing detected a BRAF V600E mutation using the CE-IVD certified PNAClamp^™^ BRAF Mutation Detection Kit (HLP Panagene Co., Ltd.), performed according to the manufacturer's instructions. No epileptiform activity was observed on preoperative electroencephalography or intraoperative electrocorticography.

Due to interval progression, a second craniotomy was undertaken in January 2024, to resect the right superior frontal gyrus MTS (Figs. 1B and 2Bb). Both procedures were uneventful. The patient and fetus remained clinically stable without neurological deficit, and the patient was discharged to close outpatient surveillance in February 2024.

### Initiation of targeted therapy

A follow-up MRI in February 2024 demonstrated progression of brain MTS (Fig. 2Bc and Bd) and the emergence of a new unresectable left hippocampal MTS, with perifocal edema (Fig. 2Be). At 30+1 weeks' gestation, the patient was counseled regarding three potential management strategies: i) Induction of preterm delivery to permit systemic therapy; ii) initiation of systemic therapy during pregnancy; or iii) a conservative watch-and-wait approach.

A multidisciplinary board, chaired by the hospital director and including an oncologist, neonatologist, gynecologist, neurosurgeon, neurologist and clinical psychologist, reviewed the case. Counseling addressed maternal neurological risks, potential teratogenicity of targeted therapy, expected neonatal outcomes at different gestational ages, the rationale for delaying vs. expediting delivery and the scope of fetal surveillance. The patient was given >24 h to reflect on the information and had the opportunity to consult with their husband and family.

After providing informed consent, the patient elected to continue the pregnancy and initiate systemic targeted therapy. In February 2024, oral dabrafenib (150 mg twice daily) and trametinib (2 mg once daily) were commenced (Fig. 1).

A subsequent MRI in March 2024 demonstrated favorable postoperative changes (Fig. 2Ca and Cb), along with a marked reduction in brain MTS volume compared with that in February 2024 (Fig. 2Cc-Ce).

### Peripartum events

To allow for fetal maturation, delivery was planned for ~34 weeks' gestation. Oncological therapy was interrupted 3 days beforehand, and intramuscular dexamethasone (6 mg every 12 h; four doses) was administered for fetal lung maturation.

In March 2024 (33+6 weeks), the patient experienced a focal impaired consciousness seizure, necessitating an urgent cesarean section. A eutrophic premature male neonate was delivered, weighing 2,030 g and measuring 46 cm, with Apgar scores of 9/9/9 (24). The patient resumed targeted oncological therapy postoperatively and was started on levetiracetam, titrated to 500 mg twice daily, for seizure control.

### Neonatal findings and follow-up

At birth, the neonate exhibited delayed cranial ossification with widely open anterior and posterior fontanelles and a broad sagittal suture. Cutaneous findings included fragile vasculature with dark discoloration, most pronounced in the cubital veins, and several isolated dark gray macules (4–5 mm in diameter).

The neonate developed respiratory insufficiency requiring admission to the neonatal intensive care unit (NICU) and continuous positive airway pressure support. Transient hypotension was managed with intravenous crystalloids. Progression to respiratory distress syndrome necessitated surfactant replacement and 6 days of mechanical ventilation. Pulmonary hypertension was treated with inhaled nitric oxide and vasopressors. Cranial ultrasound revealed a grade I intraventricular hemorrhage and stage I hypoxic-ischemic encephalopathy. No congenital malformations were identified. Histological examination of the placenta and umbilical cord showed no evidence of metastatic disease (Fig. S1). The neonate was discharged 27 days after birth.

The child has been followed up at the National Institute of Children's Diseases (Bratislava, Slovakia). Neurodevelopmental assessment at 11 months of chronological age (corrected age, 9 months) included standardized testing with the Bayley Scales of Infant and Toddler Development, Fourth Edition (Bayley-4) (25) for cognitive, communication and motor domains, and the Bayley-III (26) for social-emotional behavior. Cognitive function (37th percentile), communication (50th percentile) and motor function (73rd percentile) were all within the average range (16th-84th percentile) for corrected age, while social-emotional behavior was in the higher-average range (75th percentile). Repeated screening with the S-PMV test (Slovakian, *Skríning psychomotorického vývoja*) was also normal. At the latest follow-up (May 2025), growth and psychomotor development were within normal limits.

### Maternal oncological course and follow-up

The patient's targeted therapy with D + T was terminated after 7 weeks (April 2024) due to pyrexia and fatigue. The patient was subsequently transitioned to combined immune checkpoint inhibitor therapy as second-line treatment (Fig. 1). The concurrent regimen of ipilimumab (3 mg per kg) and nivolumab (1 mg per kg) was administered intravenously every 3 weeks for four doses, followed by maintenance nivolumab (240 mg) every 2 weeks.

In May 2024, the patient experienced a second epileptic seizure of the focal to bilateral tonic-clonic type, and the levetiracetam dosage was increased to 750 mg twice daily. As of May 2025, the patient remains on maintenance nivolumab therapy with partial disease remission and no evidence of treatment-related toxicity (Fig. 2Da-De).

Cognitive function remained intact, with a Montreal Cognitive Assessment (27) score of 30 out of 30. Quality of life, assessed with the Patient-Weighted Quality of Life in Epilepsy Inventory (version 2) (28), was 70.6 out of 100. Psychological evaluation revealed moderate anxiety, with a Generalized Anxiety Disorder-7 (29) score of 8 out of 21 and mild depressive symptoms, with a Patient Health Questionnaire-9 (30) score of 9 out of 27, without suicidal ideation.

The patient remains under regular oncological follow-up, receiving maintenance nivolumab every 2 weeks and under neurological surveillance every 3 months. The partial remission response could be durable as demonstrated in the CheckMate 204 study (12); however, long-term vigilance is warranted.

## Discussion

CM is a highly aggressive malignancy responsible for >60,000 mortalities annually (31). Over the past decade, innovative systemic therapies, including MAPK pathway inhibitors (BRAF and MEK) and immune checkpoint blockers (cytotoxic T-lymphocyte associated protein 4 and programmed cell death protein 1), have notably improved melanoma prognosis, with 3-year overall survival rates reaching 41.3 and 58.4%, respectively. During the first year of treatment, the combination of BRAF and MEK inhibitors has shown superior efficacy compared with immune checkpoint blockade, with a rapid onset of response even in brain MTS, although typically limited to 6 months (11,32). In patients with symptomatic brain MTS, intracranial responses to vemurafenib (a BRAF inhibitor) were seen in only 16% of cases (33). By contrast, D + T therapy achieved an intracranial response rate of 58% in patients with BRAF V600-mutant melanoma brain MTS, compared with 31% in patients treated with dabrafenib monotherapy (11).

Human placental cotyledon models indicate notable passage of D + T molecules through the placental barrier, with higher fetal transfer for dabrafenib (14.9%) compared with trametinib (8.6%) (34). Preclinical animal studies have shown that BRAF inhibitors (vemurafenib, dabrafenib and encorafenib) possess teratogenic potential, while trametinib (a MEK inhibitor) has been associated with possible teratogenic and embryotoxic effects (35). Dabrafenib demonstrated teratogenic and embryotoxic properties at doses three times higher compared with standard human exposure (36,37). D + T may disrupt fetal growth and development by inhibiting the RAS/MAPK signaling pathway, potentially causing congenital defects (including cardiomyopathies, and facial and skeletal anomalies), developmental delay, intellectual disability and tumor predisposition (35,38). Furthermore, the MAPK/ERK pathway serves a crucial role in trophoblast proliferation, making BRAF/MEK inhibitor use particularly concerning during early pregnancy (5,39).

As of May 2025, to the best of our knowledge, no reports have documented the combined use of BRAF and MEK inhibitors during pregnancy. Available data and well-controlled studies on the safety of D + T in pregnant women remain limited, precluding definitive conclusions (35). Current European Society for Medical Oncology (ESMO) and American Society of Clinical Oncology guidelines advise against combined BRAF/MEK inhibition during pregnancy due to teratogenic and embryotoxic risks. As alternatives, interferon-α or BRAF inhibitor monotherapy may be considered as temporizing measures if urgent systemic treatment cannot be delayed (40–43).

In the present case of advanced metastatic melanoma during pregnancy, two surgical resections of the largest symptomatic brain MTS were prioritized. However, due to further inoperable progression and the patient's refusal of preterm delivery, systemic therapy with D + T was initiated, based on its superior efficacy over vemurafenib or dabrafenib monotherapy (11,33,44). The immediacy of life-threatening brain MTS justified short-term dual BRAF/MEK inhibition despite guideline cautions, supported by multidisciplinary consensus, comprehensive maternal counseling and close materno-fetal monitoring. Therapy was initiated only after the period of fetal organogenesis had been completed. Histological examination of the placenta and umbilical cord, in line with ESMO guidance, revealed no metastatic involvement.

There are isolated reports of BRAF inhibitor use in pregnancy-associated stage IV melanoma (Table I). In all 4 cases (including one set of twins), vemurafenib was administered (14–17), with low-level transplacental transfer (37). Administration occurred between 17 and 25 weeks' gestation, and all 5 infants were born prematurely (26–36 weeks). A total of 3 had a low birth weight (1,028, 950 and 900 g), 1 weighed 2,510 g and 1 had an unspecified weight, with 3 of the newborns requiring NICU admission. With regard to the mothers, 1 patient with a solitary brain metastasis in the temporal lobe died from an intracranial hemorrhage 78 days after treatment initiation (16). Another was diagnosed with cerebral and dural MTS postpartum and died 3.5 months after initiating vemurafenib therapy (15). The present case therefore contrasts with previous vemurafenib-only reports, being the first to describe combined BRAF/MEK inhibition in pregnancy. Notably, the patient remains alive in partial remission at >14 months (452 days) after the initiation of D + T.

Checkpoint inhibitors introduced postpartum in the present case provided additional long-term disease control. Previous research highlights the heterogeneity of the melanoma immune microenvironment and identifies a prognostic NOD-like receptor gene signature strongly associated with survival in skin CM (45). Such insights into tumor-immune interactions may help explain the durable partial remission observed in the present patient under nivolumab maintenance.

The neonate in the present case exhibited delayed cranial ossification, fragile vasculature and respiratory complications requiring admission to the NICU. These findings are most likely multifactorial, reflecting prematurity and possible *in utero* exposure to targeted therapy. While inhibition of the MAPK pathway provides a plausible mechanistic link to abnormal ossification, causality cannot be established. A limitation is that therapeutic drug monitoring of D + T plasma levels was not available at the Academician Ladislav Dérer Hospital (University Hospital Bratislava, Bratislava, Slovakia) and therefore was not performed.

The present case provides rare insight into the management of PAM with brain MTS, illustrating that, in selected situations with immediate maternal risk, combined BRAF/MEK inhibition may be justified following multidisciplinary evaluation, despite current guideline cautions. The present report emphasizes the importance of individualized decision-making, careful materno-fetal monitoring and long-term follow-up of both mother and child.

## Supplementary Material

Supporting Data

## Figures and Tables

**Table I.** Case reports of targeted therapy in pregnancy-associated stage IV melanoma.

First author, year	Drug	Patient age, years	Weeks gestation at initiation of treatment	Weeks gestation at delivery	Neonate birth weight, g	NICU admission of neonate	Metastatic involvement in patient	(Refs.)
Pagan *et al*, 2019	Vemurafenib	25	25	34	2,510	Yes	Lungs, subcutaneous and lymph node	(14)
Maleka *et al*, 2013	Vemurafenib	37	25	30	1,028	No	Subcutaneous, liver, lungs, brain and meninges	(15)
de Haan *et al*, 2018	Vemurafenib	30	22	26	950 and 900	Yes (both)	Cutaneous, bowel, mesentery, breast and brain	(16)
Marcé *et al*, 2019	Vemurafenib	29	17	36	Not mentioned	No	Liver, lymph node, subcutaneous and bone	(17)
Present case	Dabrafenib + trametinib	35	30	33	2,030	Yes	Brain	

NICU, neonatal intensive care unit.

## Data Availability

The data generated in the present study may be requested from the corresponding author.

## Abbreviations

BRAF: B-Raf proto-oncogene serine/threonine kinase
CM: cutaneous melanoma
D + T: dabrafenib and trametinib
MAPK: mitogen-activated protein kinase
MEK: mitogen-activated protein kinase kinase
MTS: metastases
NICU: neonatal intensive care unit
PAM: pregnancy-associated melanoma
WB: whole-body
