# Supplementary material for: Combined BRAF/MEK inhibition for BRAF-mutant melanoma brain metastases in pregnancy: A case report
Source: Oncol Lett. 2025 Oct 24;31(1):8. doi: 10.3892/ol.2025.15361 (PMC12592975; doi:10.3892/ol.2025.15361)

Figure S1. Histopathology and immunohistochemistry of the resected temporal metastasis, placenta and umbilical cord. (A and B) H&E staining of the left temporal melanoma metastasis (magnification, x200). Immunohistochemistry of the temporal metastasis showing (C) cytokeratin AE1/3(-), (D) S100(+), (E) SOX10(+), (F) melan-A(+), (G) HMB-45(+), (H) preferentially expressed antigen in melanoma(+) results and (I) a Ki-67 proliferation index up to 30% (magnification, x200). (J and K) H&E staining of the placenta showing no metastatic involvement (magnification, x100). (L) H&E staining of the umbilical cord showing no metastatic involvement (magnification, x4). -, negative; +, positive; H&E, hematoxylin and eosin.

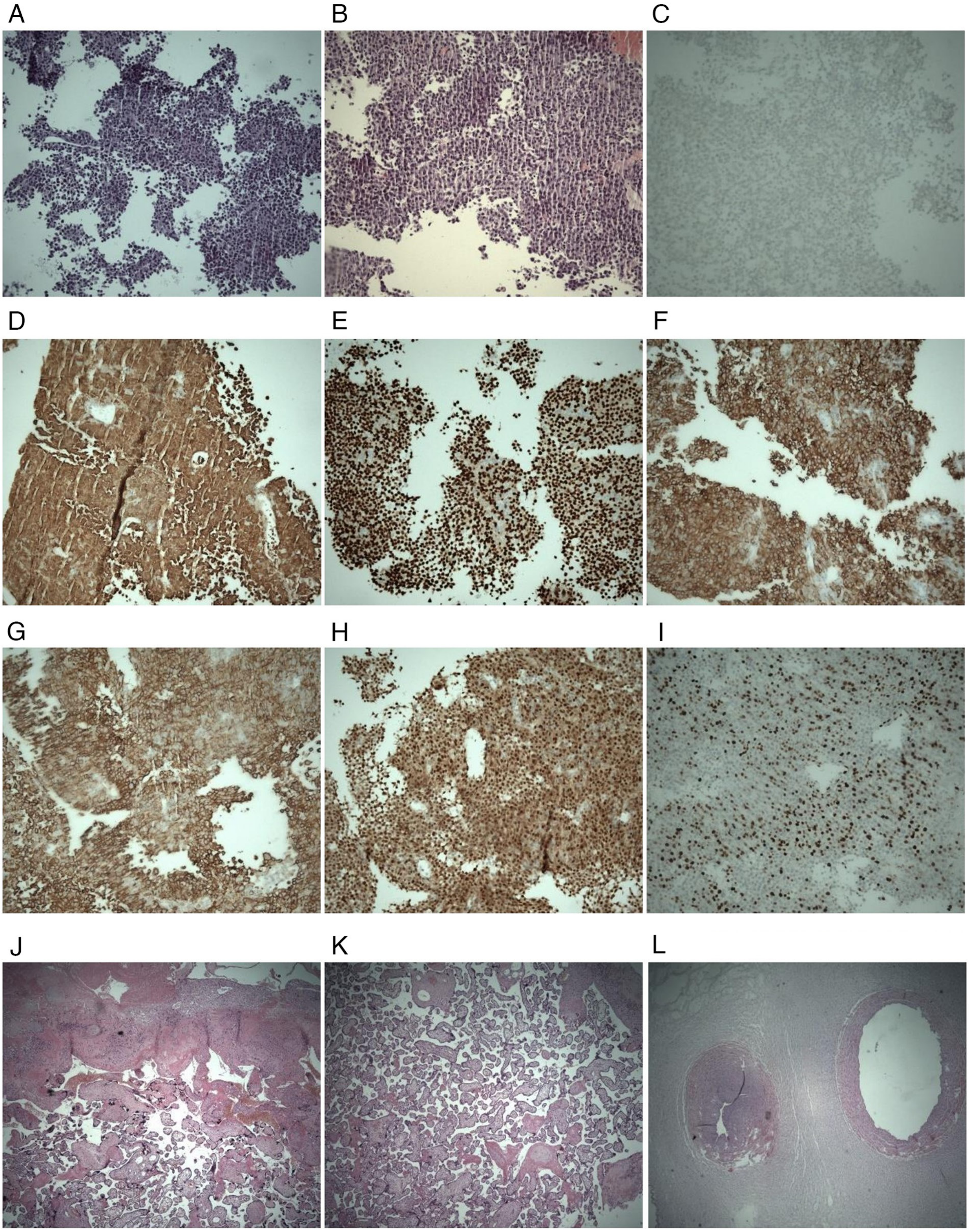

Supplement: Supporting Data [file Supplementary_Data.pdf]
